# Stance controlled knee flexion improves stimulation driven walking after spinal cord injury

**DOI:** 10.1186/1743-0003-10-68

**Published:** 2013-07-04

**Authors:** Thomas C Bulea, Rudi Kobetic, Musa L Audu, Ronald J Triolo

**Affiliations:** 1 Department of Biomedical Engineering, Case Western Reserve University, Cleveland, OH, USA; 2 Motion Study Laboratory, Louis Stokes Cleveland Department of Veterans Affairs Medical Center, Cleveland, OH, USA; 3 Department of Orthopaedics, Case Western Reserve University, Cleveland, OH, USA

**Keywords:** Functional neuromuscular stimulation, Hybrid neuroprosthesis, Controllable orthosis, Gait, Spinal cord injury, Gait, Exoskeleton

## Abstract

**Background:**

Functional neuromuscular stimulation (FNS) restores walking function after paralysis from spinal cord injury via electrical activation of muscles in a coordinated fashion. Combining FNS with a controllable orthosis to create a hybrid neuroprosthesis (HNP) has the potential to extend walking distance and time by mechanically locking the knee joint during stance to allow knee extensor muscle to rest with stimulation turned off. Recent efforts have focused on creating advanced HNPs which couple joint motion (e.g., hip and knee or knee and ankle) to improve joint coordination during swing phase while maintaining a stiff-leg during stance phase.

**Methods:**

The goal of this study was to investigate the effects of incorporating stance controlled knee flexion during loading response and pre-swing phases on restored gait. Knee control in the HNP was achieved by a specially designed variable impedance knee mechanism (VIKM). One subject with a T7 level spinal cord injury was enrolled and served as his own control in examining two techniques to restore level over-ground walking: FNS-only (which retained a stiff knee during stance) and VIKM-HNP (which allowed controlled knee motion during stance). The stimulation pattern driving the walking motion remained the same for both techniques; the only difference was that knee extensor stimulation was constant during stance with FNS-only and modulated together with the VIKM to control knee motion during stance with VIKM-HNP.

**Results:**

Stance phase knee angle was more natural during VIKM-HNP gait while knee hyperextension persisted during stiff-legged FNS-only walking. During loading response phase, vertical ground reaction force was less impulsive and instantaneous gait speed was increased with VIKM-HNP, suggesting that knee flexion assisted in weight transfer to the leading limb. Enhanced knee flexion during pre-swing phase also aided flexion during swing, especially when response to stimulation was compromised.

**Conclusions:**

These results show the potential advantages of incorporating stance controlled knee flexion into a hybrid neuroprosthesis for walking. The addition of such control to FNS driven walking could also enable non-level walking tasks such as uneven terrain, slope navigation and stair descent where controlled knee flexion during weight bearing is critical.

## Background

Approximately 275,000 people are living with traumatic spinal cord injury (SCI) in the U.S. [1], and regaining walking mobility is routinely identified as a chief priority by this population [2]. Functional neuromuscular stimulation (FNS) can be used to restore walking after SCI via electrical activation of paralyzed muscles. Restoring gait after paralysis with FNS has been shown to improve overall health, including increased cardiovascular fitness, improved muscle strength and blood flow, better bladder and bowl function, reduced muscle spasticity, reduction of pressure wounds, and decreased incidence of depression [3-8]. The original strategy for FNS walking elicited flexor-withdrawal reflex for swing phase flexion through stimulation of the peroneal nerve while stimulation of quadriceps provided knee extension for stance phase [9]. Direct targeting of individual muscles expanded FNS gait by providing isolated actions at individual joints, such as knee extension and flexion, allowing synthesis of walking motion with a near normal gait cycle [10]. A 16 channel FNS system using this targeted approach could reliably restore walking over short distances limited by quadriceps fatigue [11]. Long term use of FNS systems for walking exercise spanning 17 years was reported [7].

Combining FNS with a lower extremity orthosis to create a hybrid neuroprosthesis (HNP) offers the opportunity to improve gait efficiency by limiting stimulation to limb propulsion for forward progression, while utilizing stability of a locked brace to prevent collapse. The early HNP approach coupled hip extensor stimulation with a reciprocating gait orthosis (RGO) while keeping the knees locked [12-14]. HNPs incorporating controllable knee joint mechanisms that unlock the knee for swing motion while retaining stability for body weight support during stance were reported to reduce the amount of stimulation required for gait as compared to FNS systems alone [15-18]. Recent efforts in the HNP field have incorporated controllable devices to assist in limb maneuvers beyond locking the knee in stance phase. The spring-brake orthosis (SBO) stored energy from knee extensors to aid in swing phase hip and knee flexion [19]. The controlled-brake orthosis (CBO) shaped knee and hip trajectories using magnetic particle brakes [20]. An HNP which coupled knee flexion with ankle dorsiflexion during swing reduced compensatory mechanisms during gait [21]. The energy storing orthosis transferred excess energy from quadriceps stimulation to aid in ipsilateral hip extension for forward progression [22]. A joint coupled orthosis (JCO), which couples knee and hip flexion during swing, was designed for walking in individuals with SCI [23]. In addition to energetically passive orthoses, several HNPs have been developed which incorporate powered exoskeletons to assist FNS driven joint motion, including the hybrid assistive system [24]. A recent surge in exoskeleton technology has resulted in numerous robotic devices for restoring mobility after paralysis [25] however these systems do not incorporate FNS for walking and are beyond the scope of this study.

In FNS-driven walking, the knee remains extended for the majority of stance phase because the eccentric contractions required to regulate flexion under loading are not readily attained or controlled with stimulation [26]. Similarly, knee locking mechanisms common to most HNPs prevent knee motion during stance phase. In normal gait, knee flexion during loading response provides valuable shock absorption, stabilizes the knee, and contributes to preserving forward progression [27]. Also, knee flexion during terminal stance and pre-swing is critical for toe clearance and center of mass progression [27,28]. Knee flexion during stance phase of normal walking does not significantly reduce vertical motion of center of mass [29] yet HNP gait is constrained by an orthosis, which limits motion to the sagittal plane and locks the ankle joint in a neutral position. These constraints consign a significant role in maintenance of forward momentum and control of center of mass vertical motion to stance phase knee motion.

Considerable evidence suggests that allowing some stance phase knee flexion during pathological gait is beneficial. In studies of healthy individuals, knee flexion during loading response and weight acceptance is less than 10° at slower gait speeds typically observed in FNS walking (0.5 m/sec) [30]. But unlike normal gait, the effects of a stiff knee on the leading limb (i.e. during loading response and mid-stance) are compounded in HNP walking by the use of a walker, ankle locking by AFO, and orthotic braces which constrain limb motion to the sagittal plane. Stimulated or locked quadriceps immediately after foot contact results in knee hyperextension, which limits shock absorption [31]. Stiff leading limb during FNS gait persists through loading response phase causing hip flexion and trunk tilt toward the walking aid, an action which impedes forward progression of body center of mass [31]. During mid-stance, stiff limb restricted to the sagittal plane creates a compass type gait causing excessive vertical center of mass motion that requires excessive trunk and upper extremity effort to carry the body over the stance limb [31]. During late stance (terminal stance and pre-swing), stiff knee has implications for knee flexion and forward progression during swing phase. Study of stiff legged gait has determined that delayed deactivation of knee extensors during late stance reduces peak knee flexion [32]. Furthermore, reduced knee flexion velocity at toe-off has been identified as a key cause of limited knee flexion during swing phase [33,34], and delayed deactivation of knee extensor muscles can significantly diminish knee flexion velocity at toe-off [35]. These studies [32-35] examined implication of stiff knee at toe-off for gait speeds ranging from 0.9-1.4 m/sec, which can be faster than FNS-driven gait. But unlike loading response, knee flexion during pre-swing does not diminish with decreased gait speed [30]. In FNS-only walking, turning knee extensor stimulation off before the leg is fully unloaded can lead to collapse [31]. Delayed deactivation of knee extensor stimulation creates residual tone in pre-swing that hinders knee flexion in early swing, leading to toe-drag and instability.

The results of the above studies suggest that FNS and HNP walking systems which retain a stiff knee through muscle activation or mechanical locking impede weight transfer and forward progression by limiting knee motion during stance phase. The purpose of this investigation was to compare stiff legged FNS-driven gait with a new HNP capable of providing stance phase knee motion regulated by a variable impedance knee mechanism (VIKM-HNP) [36-38]. In this study we evaluated the hypothesis that by controlling stance phase knee motion, the VIKM-HNP system can provide a more natural gait compared to FNS systems which retain a stiff knee joint. The primary outcome measure examined in this study was knee angle, with secondary measures of ground reaction force and knee joint moment. These quantities were measured during walking with FNS-only and with VIKM-HNP. Furthermore, we examined effects of these quantities on changes in gait speed and step length.

## Methods

The objective of this study was to compare walking function generated by FNS-only (control case) with that provided by the VIKM-HNP system (experimental case). We have previously reported design and testing of a variable impedance knee mechanism (VIKM) capable of controlling knee motion [36-38]. The VIKM uses a passive, controllable damper to provide up to 64.5 Nm of torque to resist and control knee motion, with a response time of less than 35 ms. The VIKM can lock the knee to prevent motion and can regulate knee flexion while supporting body weight. The VIKM was incorporated into a hybrid neuroprosthesis (VIKM-HNP) which allowed unencumbered motion at the hips under FNS control while locking the ankle joint in a neutral position with ankle foot orthosis (AFO). Total weight of the VIKM-HNP exoskeleton, including battery and damper driving and sensor processing electronics was approximately 11.1 kg. Each VIKM mechanism including uprights and four-bar linkage knee joint weighs approximately 3.5 kg, with 0.9 kg for the damper. The weight of the prototype VIKM-HNP was not optimized and is increased by the use of off-the-shelf components. Next, we developed a closed loop control system, featuring a finite state machine, for walking using the VIKM-HNP [37,38]. The control system modulated knee extensor stimulation and activated the VIKM to control knee motion during stance phase. Potentiometers measuring knee angle and force sensitive resistors under the foot measuring heel and toe contact were used to segment the gait cycle into five discrete phases: loading response, mid-stance, terminal stance, pre-swing, and swing. Knee extensor stimulation was turned off during loading response, terminal stance, and pre-swing phases. Knee flexion, controlled by the VIKM, was allowed during loading response and pre-swing phases. The knee was locked by the VIKM during terminal stance. A proportional controller based on knee angular velocity was used to control rate of knee flexion by means of VIKM resistance. Knee flexion of up to 16° in loading response and up to 40° in pre-swing was allowed [38]. During mid-stance phase, a short burst of knee extensor stimulation was applied to extend the knee to neutral following loading response to provide clearance for the contralateral swing leg. This burst of stimulation was identical to knee extensor stimulation provided during mid-stance phase in FNS-only walking. Any flexion of the knee in this phase was resisted by the VIKM. The VIKM remained off during swing phase while knee motion was under FNS control.

One male volunteer with thoracic (T7) level SCI categorized by the American Spinal Injury Association (ASIA) as ASIA A indicating complete loss of motor and sensory function below the level of injury was enrolled and consented for study participation as required by the local institutional review board. At the time of testing, he was 50 years old, 27 years post-injury, weighed 62.2 kg and measured 1.74 m in height. He was previously implanted with an FNS system comprised of 16 percutaneous intramuscular electrodes targeting muscles bilaterally for hip extension (gluteus maximums and posterior portion of adductor magnus), hip flexion (tensor fasciae latae, sartorius), knee extension (vastus medialis, lateralis, and intermedius), knee flexion (sartorius and gracilis), and ankle dorsiflexion (tibialis anterior) by activation of common peroneal nerve stimulation. The stimulation of common peroneal nerve during swing phase was included in the HNP with fixed AFO because it provided additional hip and knee flexion through the withdrawal reflex response. In addition, this isolated differences in stimulation pattern between control and experimental case to the knee joint. The participant had over 25 years of walking experience with FNS-only systems. Gait with FNS systems varies widely between individuals due to disparity in muscle strength, passive elastic properties, response to stimulation, and location of implanted electrode within each muscle. Previous studies have shown that wearing the brace portion of the HNP with joint constraints removed had minimal affect on FNS-only gait [17]. Thus, in this case study, the subject served as his own control, whereby data from walking with FNS-only system was compared with data from walking with VIKM-HNP.

An individualized baseline stimulation pattern was constructed using previously established rules for FNS gait [10]. Stimulation was delivered through percutaneous electrodes using an external control unit worn around the waist and a finger switch that allowed user control. In both control and experimental case the participant walked using the baseline stimulation pattern in an auto-triggered free cycling mode that started with the press of a finger switch and continued until the finger switch was pressed again to stop. Gait speed was controlled by linear scaling of the stimulation pattern to the participant’s preferred speed. The scaling factor remained the same across control and experimental conditions. In control case, the participant walked using the baseline stimulation pattern. In the experimental case, the participant wore the prototype VIKM-HNP orthosis and knee extensor stimulation modulation was synchronized with VIKM during loading response, terminal stance, and pre-swing phases [38]. The purpose of this stimulation modulation was twofold: allow the VIKM to control knee motion during stance phase while allowing knee extensor muscles to rest. Knee extensor stimulation was active during mid-stance phase to bring the knee back to full extension. The hip joints were unconstrained and under control of the preprogrammed FNS pattern for both conditions. The ankle joints were locked in a neutral position using AFOs and ankle joint muscle stimulation did not vary from the baseline pattern. This configuration allowed evaluation of the effects produced by the altered knee joint control provided by the HNP-VIKM to be compared to FNS-only walking.

A 16 camera Vicon MX 40 motion capture system (Vicon, Inc., Oxford, UK) operating at 200 Hz and a set of 21 reflective markers, specifically placed to accommodate the brace [39], were used to quantify motion of the lower extremity and trunk segment. Two force platforms (AMTI Inc., Watertown, MA) were used to measure ground reaction force vectors (GRFs) at 1000 Hz. A walker instrumented with two AMTI load cells sampled at 200 Hz measured the vertical support provided by the upper extremities during ambulation. During each trial, the subject was asked to walk approximately 10 meters at his previously determined preferred pace using the automated walking pattern described above. A spotter remained close to the participant to assure safety. The first and last two steps of each trial were discarded to ensure only steady state walking was analyzed. Data from FNS-only walking (control) were collected over two sessions on different days for a total of 38 strides; data from VIKM-HNP walking (experimental) were collected over three sessions on different days for a total of 30 strides.

Kinematic and kinetic data were low pass filtered using 5^th^ order, zero-phase Butterworth filters with cutoff frequencies of 5 Hz and 20 Hz, respectively. The instantaneous gait speed, an indicator of forward progression [27], was measured as the velocity of the center point of the pelvis. External knee joint moment in the plane of progression was calculated by inverse dynamics using inertia properties calculated from the geometry of the VIKM orthosis and measurements of the participant’s limbs and weight. Kinetic data (GRFs and external knee joint moment) were decimated to match the kinematic sampling rate, and all data were averaged (± 1 standard deviation) with respect to percentage gait cycle, defined as heel strike to heel strike identified by local minimum of heel markers in the vertical axis [40]. The kinematics (e.g. joint angles) of each leg were analyzed independently because of the distinct response to baseline stimulation. Unlike kinematic data, kinetic data from left and right limbs were combined for analysis. This combination was permissible because force data were analyzed independent from kinematic data, with the exception of knee moment; however, knee moment was expressly analyzed during stance phase, when left and right knee trajectory was observed to be similar within each condition. For comparison between experimental conditions, a one factor repeated analysis of variance (ANOVA) with a 95% confidence interval (*p* < 0.05) was used to determine statistical significance.

## Results

### Kinematics

**Figure 1:**
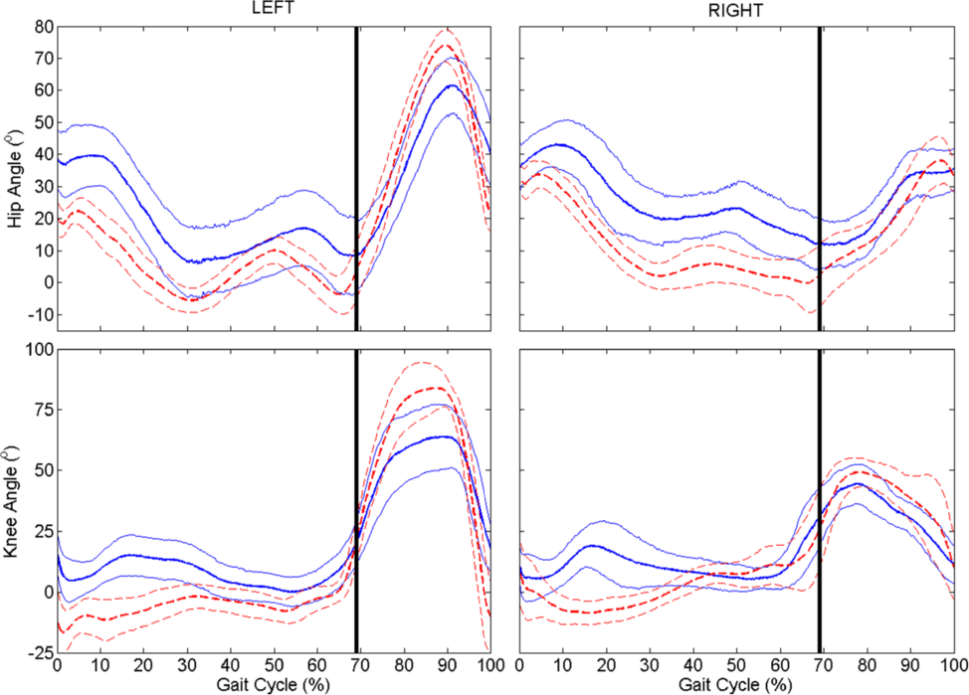
**Average (± 1 std. dev.) sagittal plane hip and knee angles across experimental conditions.** Solid line is VIKM-HNP, dash line FNS-only; flexion is positive. Vertical black line indicates transition from stance to swing phase.

**Figure 2:**
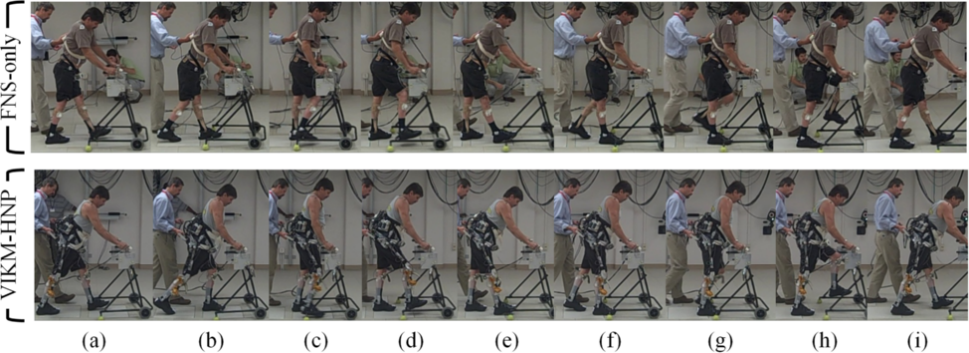
**Stride by stride (left heel strike to left heel strike) comparison of walking restored under control and experimental conditions.(a)** left (L) initial contact, right (R) terminal stance, **(b)** L loading response, R pre-swing, **(c)** L mid-stance, R mid-swing, **(d)** L terminal stance, R terminal swing, **(e)** L terminal stance, R initial contact, **(f)** L pre-swing, R loading response, **(g)** L mid-swing, R mid-stance, **(h)** L terminal swing, R terminal stance, **(i)** L initial contact, (R) terminal stance.

**Figure 3:**
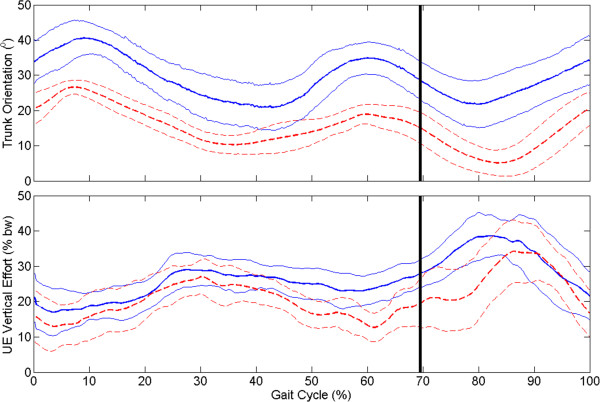
**Average (± 1 std. dev.) sagittal plane trunk orientation and upper extremity (UE) vertical force across experimental conditions: VIKM-HNP (solid line) and FNS-only (dashed line).** Trunk orientation of 0° is vertical, positive is forward tilt. UE force was normalized by user body weight. Vertical black line indicates transition from stance to swing phase.

A significant change in sagittal plane knee angle during stance phase was observed with the VIKM-HNP compared to FNS-only for both the left and right limbs (Figure 1, bottom row). The differences are most pronounced during loading response and pre-swing phases (Figure 2b&f). Loading response characterized by knee flexion is present with the VIKM-HNP, with the peak average knee angle for left (15°) and right (19°) limb occurring at approximately the same point in the gait cycle (17%). In FNS-only gait the knee remained hyper-extended (less than 0°) during early to mid-stance (< 50% GC), with peak average knee angle of 1° (L) and 9° (R) occurring much later in stance (31% (L), 46% (R)) than with VIKM-HNP. Stance phase hip was more flexed for both limbs during walking with VIKM-HNP compared to FNS-only (Figure 1, top row) due to increased trunk lean during the experimental condition (Figure 3, top). Average trunk orientation was significantly less vertical (*p* < 0.001) with the VIKM-HNP, while normalized upper extremity (UE) force averaged over the gait cycle (Figure 3, bottom) was slightly, but not significantly (*p* = 0.13), elevated. The trunk range of motion during gait was similar across conditions implying a similar amount of UE force on the walker was necessary to support the trunk in both cases.

**Figure 4:**
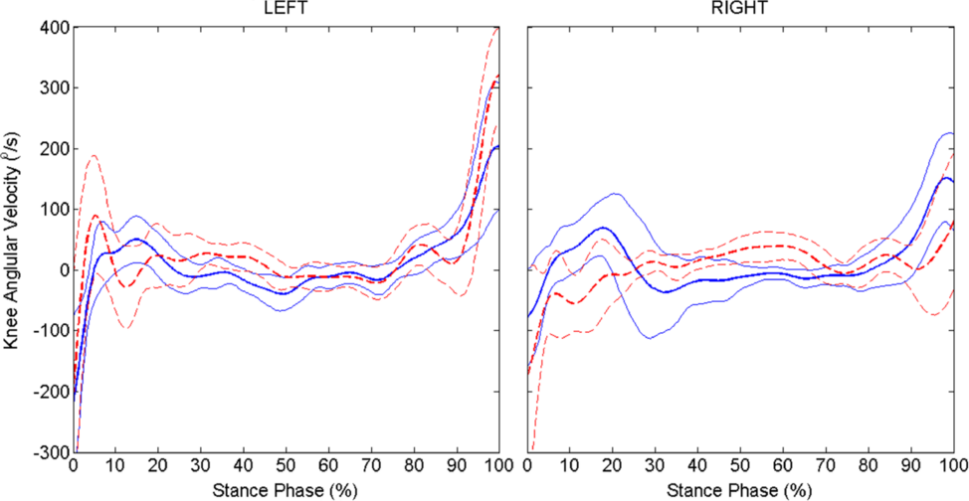
Average (± 1 std. dev.) knee angular velocity during stance phase across experimental conditions: VIKM-HNP (solid line) and FNS-only (dashed line).

Left and right limb trajectories were different during swing phase (Figure 1). The response of left limb to hip and knee flexor stimulation was much stronger resulting in more swing phase hip and knee flexion under both conditions than the right side. Left limb average maximum hip and knee angles during swing were significantly less with VIKM-HNP than FNS-only (Table 1), an effect which is likely due to weight of the VIKM. Interestingly, right limb average maximum hip and knee angles during swing were not significantly different between conditions (Table 1). During FNS-only walking, left knee flexion velocity at toe-off is greater than the right (Figure 4), and accordingly peak left knee angle is greater during swing (Table 1). The weight of the VIKM orthosis appeared to decrease peak flexion velocity in the stronger left limb, reducing swing phase knee flexion. On the right limb, knee flexion began earlier in pre-swing with the VIKM-HNP, increasing flexion velocity at toe-off compared to FNS-only (Figure 4), resulting in similar swing phase flexion.

**Table 1:** Average maximum swing phase hip and knee angles across walking conditions

**Experimental condition**	**Maximum swing phase hip angle (°) (SD)**		**Maximum swing phase knee angle (°) (SD)**	
	**Left**	**Right**	**Left**	**Right**
VIKM-HNP	**64.1**	39.3	**68.1**	47.2
	**(8.4)**	(7.2)	**(12.0)**	(7.8)
FNS-Only	**74.7**	40.2	**86.9**	52.0
	**(4.8)**	(6.9)	**(8.4)**	(6.1)
p-value	**< 0.0001**	0.6346	**< 0.0001**	0.1050

Bold columns indicate differences which were statistically significant.

### Kinetics

**Figure 5:**
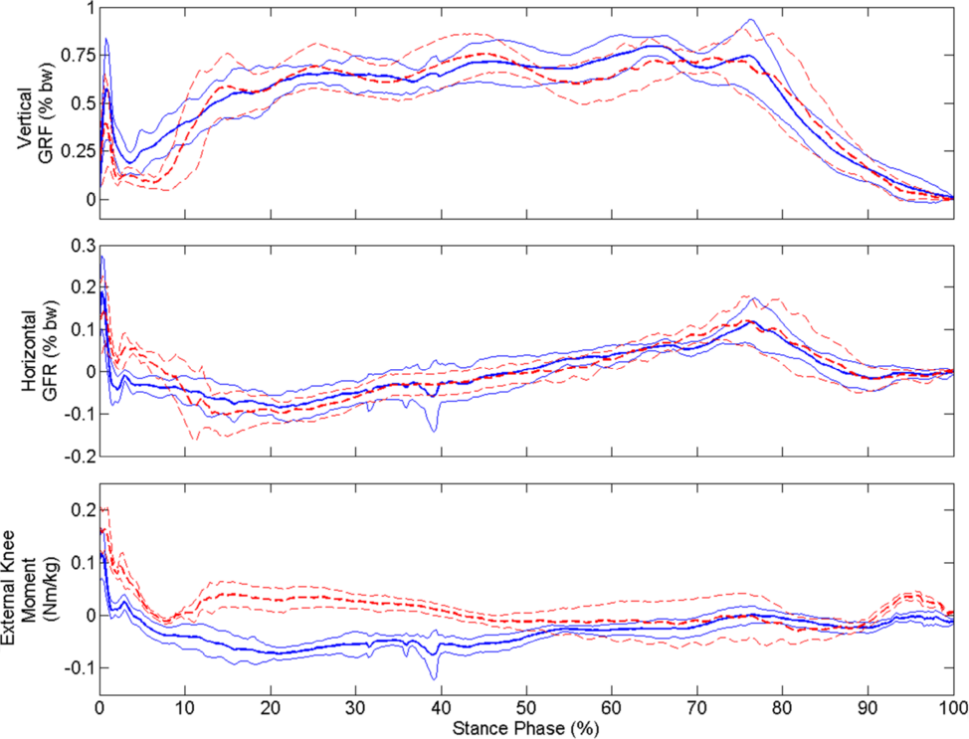
**Average (± 1 std. dev.) vertical and horizontal ground reaction forces (GRFs) and external knee moment across experimental conditions: VIKM-HNP (solid line) and FNS-only (dashed line).** GRFs (given as percentage body weight) and external knee moment (Nm/kg) are normalized by body weight and body weight plus weight of the VIKM orthosis for FNS-only and VIKM-HNP conditions, respectively. Positive values of external moment are extension, negative are flexion.

As with UE forces, average maximum vertical and horizontal GRFs were similar (*p* = 0.93, *p* = 0.77) between conditions. However, key differences were observed in GRF profile during loading response phase (Figure 5, top two rows). A large impulsive force in the vertical direction is observed under both conditions immediately following foot contact (0-2% of stance phase), which was followed by a period of weight transfer to the leading limb (2-20% of stance phase). In the FNS-only condition, weight transfer is delayed and more impulsive compared to VIKM-HNP as indicated by the slope of average vertical GRF. Horizontal GRF remained positive during the delayed vertical loading period for FNS-only, indicating force from the leading limb is acting in the opposite direction of forward progression. A faster transition to negative horizontal GRF with VIKM-HNP coincident with steady increase in vertical GRF suggests better weight transfer to the leading limb. External knee moment was significantly different between conditions (Figure 5, bottom), especially during early stance phase (< 50%). The FNS-only moment remained positive, creating extensor torque about the knee causing hyperextension during loading response and mid-stance phases (Figure 1 and Figure 2a-c). Conversely, knee moment was flexor with the VIKM-HNP during this phase, resulting in knee flexion regulated by the active VIKM orthosis. Knee extension during mid-stance phase with the VIKM-HNP (15-40% GC, Figure 1) occurred due to reactivation of knee extensor stimulation, which counteracted the external flexion moment (20-55% of stance phase, Figure 5). External knee moment during pre-swing phase (85-100% of stance phase, Figure 5) was extensor for FNS-only while it was slightly flexor for VIKM-HNP. Thus, the knee began flexing sooner and at a higher velocity in VIKM-HNP compared to FNS-only (Figure 4).

### Spatiotemporal

Average gait speed was similar across conditions; however, instantaneous gait speed was less variable with VIKM-HNP than FNS-only as measured by coefficient of variance (Table 2). Average minimum instantaneous gait speed – measured as the velocity of the pelvis center of mass – during a stride was significantly increased in the experimental case, while maximum instantaneous gait speed was similar across conditions (Table 2). Minimum instantaneous gait speed was observed immediately after foot contact under both conditions. With FNS-only, knee hyperextension during loading response resulted in temporary stalling – or backward motion – of the center of mass as indicated by the negative instantaneous speed. With VIKM-HNP, knee flexion (Figure 1), more immediate weight transfer to leading limb (Figure 5, top), and forward motion of leading limb (Figure 5, middle) may have aided in maintaining higher instantaneous gait speed during loading response. Enhanced hip flexion at the end of left swing may have contributed to increased left step length. A similar effect was not observed on the right side, likely due to limited hip and knee flexion during swing phase.

**Table 2:** Spatiotemporal parameters averaged across experimental conditions. Bold columns indicate a statistically significant difference across conditions

**Experimental condition**	Instantaneous gait speed (m/s)*		**Step length (m)**		**Gait speed (m/s)**	Coefficient of variance**
	**Minimum**	**Maximum**	**Left**	**Right**		
VIKM-HNP	**0.09**	0.73	**0.52**	0.39	0.38	0.26
FNS-Only	**−0.01**	0.72	**0.46**	0.40	0.35	0.60
p-value	**0.0275**	0.8224	**0.0025**	0.6669	0.1001	-

*Instantaneous gait speed was measured as the forward velocity of the center point of the pelvis.

**Coefficient of variation is computed from the instantaneous gait speed, averaged over the gait cycle.

## Discussion

In this case study, stance phase knee flexion was closer to normal during walking with the VIKM-HNP compared to stiff-legged FNS-only gait. The distinct presence of a loading response phase characterized by knee flexion was observed with the VIKM-HNP but not in FNS-only walking. We also observed differences across conditions in GRF during this gait phase. Both FNS-only and VIKM-HNP contained impulsive forces in the first 2% of stance (Figure 5). The profile of these forces is similar across conditions and represents the ballistic landing of the foot on the force plate (Figure 2a). This similarity is expected because the stimulation delivered during the end of swing phase is the same across conditions [38]. After initial contact, the GRF profiles differ across conditions. In VIKM-HNP, more gradual increase in the vertical component of GRF profile was observed during weight acceptance (2-20% of stance phase) than with FNS-only (Figure 5). The direction of the horizontal GRF was also different between conditions during this phase (Figure 5). These changes occurred simultaneously with increased knee flexion (Figure 1) and knee flexion velocity (Figure 4). To study the effect of these changes on forward progression, we examined the instantaneous speed of the pelvis. We observed that the minimum instantaneous speed occurred during loading response phase under both conditions. However, with VIKM-HNP minimum instantaneous speed was significantly increased compared to FNS-only (Table 2). In fact, during stiff-legged FNS-only walking instantaneous velocity of the pelvis was negative (backward) while it was positive during walking with the VIKM-HNP, indicating enhanced forward progression during this phase. The increase in instantaneous speed resulted in a small, but not statistically significant (*p* = 0.1001), increase in average gait speed. The likely reason the increase did not translate to average gait speed is because primary propulsive forces (i.e. hip extension and hip flexion by FNS) were identical across conditions, and thus, forward velocity was comparable in other phases of the gait cycle. Yet, the observed changes in GRF and instantaneous velocity of the pelvis during loading response suggest that forward progression during this phase was enhanced with VIKM-HNP compared to FNS-only.

Proper control of knee flexion during pre-swing phase is difficult to achieve in FNS-only gait, as evidenced by the difference in pre-swing flexion velocity in left and right limbs (Figure 4). Swing phase is initiated by ramping down stimulation of knee extensor muscles (quadriceps) simultaneous with initiation of knee flexor muscle stimulation (gracilis and sartorius); thus, knee flexion at toe-off is dependent on relaxation properties of knee extensors and contractile properties of knee flexors. Conversely, when the VIKM-HNP is activated to control knee flexion consistent knee flexion velocity is observed for both limbs at the end of stance (Figure 4). Knee flexion velocity at toe-off has been previously identified as a key factor for improving knee flexion during swing phase of stiff-legged gait [33]. Our results support this conclusion. In FNS-only gait, residual knee extensor moment during quadriceps relaxation resists knee flexion during pre-swing [31]. Accordingly, knee flexion was delayed with FNS-only (Figures 1 and 4). For a limb with strong flexor response to stimulation, such as the left limb in this case study, this is not a concern as sufficient knee flexion is reached. When flexor muscle contractions are not as strong - as observed in the right limb - knee flexors cannot achieve peak flexion velocity prior to toe-off (Figure 4) which can have detrimental effects on swing flexion. Such effects are prescient for FNS-driven walking utilizing surface stimulation, which have a difficult time activating non-superficial knee flexor muscles [31]. In the experimental case, knee flexion is regulated by the VIKM orthosis and knee extensor muscles are not stimulated during pre-swing phase, resulting in increased knee flexion during this phase (60-70% GC). As a result, peak knee flexion velocity is reached just prior to toe-off in the right limb, enabling knee flexion similar to FNS-only condition despite the extra weight of the orthosis (Figure 1). This result suggests VIKM-HNP control of knee motion during pre-swing phase could aid in swing limb flexion when flexor muscle strength is compromised. Across both limbs, swing phase hip flexion was similar or only slightly reduced with VIKM-HNP compared to FNS-only, suggesting that FNS-driven walking with an unconstrained brace is similar to FNS-only, a result that agrees with previous work [17].

External knee moment, which represents the loading at the knee joint, is critical to controlling knee angle during stance phase because the VIKM employs a passive damper to dissipate energy; thus, it can’t inject power into the gait cycle to regulate knee motion. External knee moment remained extensor throughout early stance phase with FNS-only (Figure 5) reinforcing knee extension created by stimulation of the quadriceps. With VIKM-HNP, external knee moment causes flexor action after initial foot contact (2% stance phase, Figure 5), enabling knee flexion (Figure 1) regulated by the VIKM with knee extensor stimulation turned off. Knee flexion persists until a short burst of stimulation is returned to knee extensors during mid-stance [38] to overcome the flexion moment and extend the knee (Figure 1) providing clearance for contralateral swing. Knee extensor stimulation intensity was identical to FNS-only walking during this phase and was enough to extend the limb, indicating that no increase in stimulation intensity (compared to FNS-only) was necessary to compensate for knee flexion. External knee moment is similar across experimental conditions during the second half of stance phase, with the exception of the final 10% (Figure 5). With FNS-only, knee extension moment and quadriceps stimulation oppose knee flexion during double support (80-100% stance phase, Figure 5, 60-70% GC, Figure 1) whereas with the VIKM-HNP the knee is able to flex passively under loading.

Most HNPs attempt to enhance the efficiency of restored walking by locking the knee joint during stance phase and unlocking during swing, reducing knee extensor stimulation requirements compared to FNS-only systems. Numerous efforts to improve HNP gait via joint coupling and coordination have emerged in recent years, yet the vast majority of HNP systems retain the stiff-legged stance phase, especially a locked knee joint, similar to FNS-only systems. Similar to HNPs which lock the knee, the VIKM-HNP system presented here turns off the knee extensors during a majority of stance phase to reduce knee extensor stimulation by 40% compared to FNS-only [38], but still allows natural stance phase knee motion (unlike locking HNPs or FNS-only) through controlled regulation of knee flexion using the orthosis. Reduced knee extensor duty cycle has the potential to delay onset of fatigue [31]. A short duration (less than 0.4 sec) of knee extensor stimulation - equal in intensity to that delivered with FNS-only - is required to extend the knee during mid-stance with the VIKM-HNP. This mid-stance knee extensor stimulation is preceded and followed by 0.4 sec and 1.1 sec of rest, respectively. This results in knee extensor stimulation duty cycle of approximately 35-40%, which has been reported to create minimal muscle fatigue [31]. Future studies outside of a motion laboratory, where continuous walking can be studied, are necessary to fully assess effects on fatigue.

In this case study, knee hyperextension after initial contact was reduced during walking with the VIKM-HNP compared to FNS-only. Coincidently, vertical ground reaction force was less impulsive during loading response, suggesting that knee flexion from VIKM-HNP may ease weight transfer. In addition, knee flexion during pre-swing phase was more consistent with the VIKM-HNP than FNS-only walking. Qualitatively, the user felt the difference between VIKM-HNP and FNS-only gait, initially reporting the experience as if his knees might be buckling giving him the feeling of falling during early stance. One session of walking with VIKM-HNP was provided to accommodate to the new gait pattern. After several trials, the user learned to trust that the VIKM would support his body weight. Following this acclimation period the user characterized walking as smoother and requiring less effort than FNS-only, though no preference was stated for VIKM-HNP or FNS-only system generally. No significant increase in UE force was observed with the VIKM-HNP, supporting the conclusion that user’s trust of the system equaled that of FNS-only. It should be noted that these results were observed in one subject walking with ankles locked by AFO during VIKM-HNP walking. Thus, no conclusion can be drawn regarding generalization of these results across individuals walking with hybrid FNS systems. However, these results provide impetus to consider inclusion of mechanisms that allow knee flexion during stance phase in future HNP designs. Our findings are in agreement with recent studies that demonstrate the benefits of including a passive, variable stiffness mechanism at the knee joint in orthoses and exoskeletons [41].

## Conclusion

The results of this study demonstrate the ability to control knee flexion during stance phase of FNS driven gait with a passive, controllable damper in one subject. Knee flexion during loading response resulted in more natural knee angle trajectory than FNS-only with less impulsive transition of weight from trailing to leading limb as measured by vertical ground reaction force. Instantaneous gait speed was also increased during this phase of gait compared to FNS-only. Controlling knee motion with the VIKM created more consistent knee flexion during pre-swing phase resulting in increased knee flexion velocity at toe-off on the weaker right limb. These results are preliminary and future work will focus on further evaluation of the VIKM-HNP system during FNS driven walking.

Stable knee flexion during weight bearing – something unattainable with FNS-only or knee locking HNPs – is essential during many community ambulation tasks beyond walking, such as descending slopes [42] or stairs [43]. Future work will also focus on implementation of the VIKM-HNP to achieve these functions.

## Abbreviations

AFO: Ankle Foot Orthosis; ASIA: American Spinal Cord Injury Association; BW: Body Weight; CBO: Controlled Brake Orthosis; FNS: Functional Neuromuscular Stimulation; GRF: Ground Reaction Force; HNP: Hybrid Neuroprosthesis; JCO: Joint Coupled Orthosis; RGO: Reciprocal Gait Orthosis; SBO: Spring Brake Orthosis; SCI: Spinal Cord Injury; UE: Upper Extremity; VIKM: Variable Impedance Knee Mechanism; VIKM-HNP: Hybrid Neuroprosthesis containing Variable Impedance Knee Mechanism.

## Competing interests

The authors declare that they have no competing interests.

## Authors’ contributions

TCB, RK, MA, and RJT collectively conceived and designed the study. TCB, RK, and MA collected data. TCB processed and analyzed data from the study. TCB, RK, MA, and RJT interpreted results from data. TCB drafted the manuscript. TCB, RK, MA, and RJT read, revised and approved the final manuscript.
